# Influence of hemoglobinopathies and glucose-6-phosphate dehydrogenase deficiency on diagnosis of diabetes by HbA1c among Tanzanian adults with and without HIV: A cross-sectional study

**DOI:** 10.1371/journal.pone.0244782

**Published:** 2020-12-31

**Authors:** Belinda Kweka, Eric Lyimo, Kidola Jeremiah, Suzanne Filteau, Andrea M. Rehman, Henrik Friis, Alphaxard Manjurano, Daniel Faurholt-Jepsen, Rikke Krogh-Madsen, George PrayGod, Douglas C. Heimburger

**Affiliations:** 1 Mwanza Research Centre, National Institute for Medical Research, Mwanza, Tanzania; 2 Faculty of Epidemiology and Population Health, London School of Hygiene and Tropical Medicine, London, United Kingdom; 3 Department of Nutrition, Exercise, and Sports, University of Copenhagen, Copenhagen, Denmark; 4 Department of Infectious Diseases, Rigshospitalet, Denmark; 5 Centre for Physical Activity Research, Rigshospitalet, University of Copenhagen, Denmark; 6 Vanderbilt Institute for Global Health and Department of Medicine, Vanderbilt University Medical Center, Nashville, TN, United States of America; University of Witwatersrand/NHLS, SOUTH AFRICA

## Abstract

**Introduction:**

Hemoglobin A1c (HbA1c) is recommended for diagnosing and monitoring diabetes. However, in people with sickle cell disease (SCD), sickle cell trait (SCT), α-thalassemia or glucose-6-phosphate dehydrogenase (G6PD) deficiency, HbA1c may underestimate the prevalence of diabetes. There are no data on the extent of this problem in sub-Saharan Africa despite having high prevalence of these red blood cell disorders.

**Methods:**

Blood samples from 431 adults in northwestern Tanzania, randomly selected from the prospective cohort study, Chronic Infections, Comorbidities and Diabetes in Africa (CICADA), were analysed for SCT/SCD, α-thalassemia and G6PD deficiency and tested for associations with the combined prevalence of prediabetes and diabetes (PD/DM) by HbA1c, using the HemoCue 501 HbA1c instrument, and by 2-hour oral glucose tolerance test (OGTT).

**Results:**

The mean age of the participants was 40.5 (SD11.6) years; 61% were females and 71% were HIV-infected. Among 431 participants, 110 (25.5%) had SCT and none had SCD. Heterozygous α-thalassemia (heterozygous α+ AT) was present in 186 (43%) of the participants, while 52 participants (12%) had homozygous α-thalassemia (homozygous α+ AT). Furthermore, 40 (9.3%) participants, all females, had heterozygous G6PD deficiency while 24 (5.6%) males and 4 (0.9%) females had hemizygous and homozygous G6PD deficiency, respectively. In adjusted analysis, participants with SCT were 85% less likely to be diagnosed with PD/DM by HbA1c compared to those without SCT (OR = 0.15, 95% CI: 0.08, 0.26, *P* < 0.001). When using OGTT, in adjusted analysis, SCT was not associated with diagnosis of PD/DM while participants with homozygous α^+^ AT and hemizygous G6PD deficiency were more likely to be diagnosed with PD/DM.

**Conclusions:**

HbA1c underestimates the prevalence of PD/DM among Tanzanian adults with SCT. Further research using other HbA1c instruments is needed to optimize HbA1c use among populations with high prevalence of hemoglobinopathies or G6PD deficiency.

## Introduction

Diabetes is becoming a major cause of morbidity and mortality in low-and middle-income countries [1]. Traditionally, diagnostics relied on random or fasting blood glucose with or without an oral glucose tolerance test (OGTT) [2]. However, OGTT testing is laborious and both OGTT and fasting plasma glucose have significant daily variability. The World Health Organization (WHO) has approved the use of glycated hemoglobin (HbA1c) as a universal diabetes test [3], as HbA1c has several advantages over OGTT: fasting is not needed, measurement is less laborious, and it estimates long-term average plasma glucose. WHO approval led to increased adoption of HbA1c for diagnosing and managing diabetes globally, including in low-and middle-income countries.

Although current assay instruments have removed many technical problems associated with the estimation of HbA1c, several challenges remain [4]. These include diagnosis and management of diabetes in patients with altered hemoglobin structure, e.g., SCD/SCT or α-thalassemia, or an enzymopathy such as G6PD deficiency.

Hemoglobin A (HbA) represents over 90% of hemoglobin in healthy red blood cells (RBCs) among normal individuals, but individuals with SCT have 30–40% as hemoglobin S (HbS) [5,6]. The presence of HbS may be associated with shorter RBC life span [7], which may reduce the time for hemoglobin glycation, leading to underestimation of glucose level by HbA1c tests. Few studies have investigated the suitability of HbA1c in diabetes diagnosis among individuals with SCD/SCT and results have been inconsistent. Two studies among African Americans found that SCT did not affect the validity of HbA1c as a diagnostic test for diabetes [6,8], while a larger cohort study showed that compared to fasting blood glucose and OGTT, HbA1c produced lower glucose equivalent estimations in individuals with SCT than in those without SCT [9].

Thalassemia is an inherited hemolytic blood disorder that involves underproduction or absence of synthesis of one or more globin chains of hemoglobin. The disorder causes hemolysis, leading to shorter RBC life span [10], resulting in a short exposure time to glucose. However, the use of HbA1c in populations with a high prevalence of thalassemia has been reported to give false high diabetes prevalence estimates as compared with other diabetes tests [11]. The two main types of thalassemia are α-thalassemia and β-thalassemia. In this study we focused on α-thalassemia because of its higher prevalence in Tanzania (37.8% heterozygous α^+^ AT and 5.2% homozygous α^+^ AT) [12] compared to β-thalassemia (0.2%) [13].

G6PD deficiency, which is common in people of Mediterranean and African origin including Tanzania [12], also predisposes RBCs to hemolysis [14] and reduces the exposure time of hemoglobin to glucose, resulting in falsely low HbA1c levels [15,16].

There are no published reports from sub-Saharan Africa on the validity of HbA1c in populations with hemoglobinopathies and G6PD deficiency, although the conditions are common. Recent studies in Tanzania found prevalence of 1.4% SCD, 15.9% to 19.7% SCT, 37.8% heterozygous α^+^ AT, 5.2% homozygous α^+^ AT, and 29.9% for combined heterozygous (G6PD(A)) and homozygous/hemizygous (G6PD(A-)) G6PD deficiency [12,13]. Given the high prevalence of disorders affecting red blood cells in Tanzania, we aimed to investigate the associations of SCT/SCD, α-thalassemia and G6PD deficiency with diagnosis of diabetes using HbA1c WHO thresholds.

We hypothesized that HbA1c tests estimate lower prevalence of PD/DM in people with SCT, α-thalassemia and G6PD deficiency than in people without these abnormalities, potentially compromising the test’s validity in these populations.

## Methods

### Ethical considerations

CICADA, including the current study procedures, received ethical approval from the Medical Research Coordinating Committee (MRCC) of the National Institute for Medical Research (NIMR) in Tanzania, from the London School of Hygiene and Tropical Medicine, and consultative approval from the National Committee on Health Research Ethics in Denmark. Oral and written information on study objectives, procedures, benefits, risks, confidentiality, and voluntary participation was provided in Swahili language. Participants’ approval to use their confidential information and stored samples, e.g., blood for the future studies, was also part of the provided information. They were also offered opportunity to ask questions which were answered to their satisfaction before they were asked to sign informed consent forms. For participants who could not read and write, solicited witnesses who were not part of the study team signed informed consent forms on their behalf.

### Study design, setting and study population

This was a cross-sectional sub-study embedded in the ongoing prospective cohort study, Chronic Infections, Comorbidities and Diabetes in Africa (CICADA), which is investigating risk factors for diabetes in adults with and without HIV (trial registration NCT03106480). CICADA enrolled 1,947 adults aged ≥ 18 years with HIV who were antiretroviral therapy (ART)-naïve, as well as ART-experienced and HIV-uninfected control participants from Mwanza, Tanzania between October 2016 and October 2017 (17). Stored blood samples collected at enrollment in CICADA were used for the current study. All CICADA participants with a stored blood sample were eligible for inclusion in this sub-study; 500 samples were selected using the simple random sampling method after assigning each participant with a random number using Microsoft Excel 2007.

### Data collection

Electronic and paper-based questionnaire data were used to gather information at enrollment into CICADA. Information on demography and socioeconomic status was gathered using the WHO STEPS questionnaire and show cards [17]. Socioeconomic status, categorised as terciles, was derived using principal components analysis based on housing characteristics, sanitation, water source, cooking fuel, ownership of electrical goods and animals, and modes of transport. Reported alcohol intake was classified as current use (within 12 months) or non-use (previous or never used), while reported smoking was classified as never, past, or currently smoking. HIV status (defined as either HIV-negative, HIV-infected/not on ART, or HIV-infected/on ART) and ART history was verified through participants’ ART cards and clinic records. Data for SCT/SCD, α-thalassemia and G6PD deficiency for this sub-study were filled in the laboratory results form and entered in Epidata.

### Anthropometry

Trained study staff assessed weight to the nearest 0.1 kg using a digital scale (Seca, Germany), and height was measured to the nearest 0.1 cm using a stadiometer fixed to the clinic wall (Seca, Germany). Anthropometric measurements were taken in triplicate, and medians were used for analysis. Body mass index (BMI) was calculated as weight (kg)/(height (m))^2^ and classified as underweight/normal (≤ 24.9 kg/m^2^) or overweight/obese (≥ 25.0 kg/m^2^) [18].

### Diabetes-related measurements

Symptoms of diabetes were solicited and recorded. Venous blood for HbA1c was drawn from those who had fasted, and participants were then given 82.5 g of dextrose monohydrate (equivalent to 75 g of anhydrous glucose) diluted in 250 ml of drinking water to drink within 5 minutes for OGTT. Blood for OGTT glucose was collected after 30 minutes and 2 hours, and glucose was measured with a point-of-care machine (HemoCue 201RT, Ängelholm, Sweden) [19]. HbA1c was measured with a point-of-care device (HemoCue HbA1c 501), which uses a boronate affinity assay to separate the glycated hemoglobin fraction from the non-glycated fraction; the instrument is calibrated to harmonize with HPLC method (HbA1c 501 Analyzer, Operating Manual).

Test results were grouped into three levels–normal, prediabetes, and diabetes using thresholds recommended by WHO [2,3]. For HbA1c, prediabetes was defined as 6.0% to < 6.5% and diabetes was defined as ≥ 6.5%; levels below 6.0% were considered normal. For OGTT, 2-hour glucose levels between 7.8 and 11.1 mmol/L were defined as prediabetes and ≥ 11.1 mmol/L indicated diabetes; levels < 7.8 mmol/L were considered normal. Prediabetes and diabetes results were then combined as one outcome, PD/DM.

### Hemoglobin assessment

Hemoglobin (Hb) levels (g/dl) were measured using a Beckman Coulter AcT5 diff AL Hematology Analyzer (Beckman Coulter, Florida, USA) and classified as anemic if Hb was ≤ 12 g/dl for females or ≤ 13 g/dl for males [20].

### DNA extraction and genotyping

Blood samples were collected in 5 ml EDTA tubes and stored at -80°C until tested. DNA was extracted from whole blood using the QIAamp DNA Mini Kit (Qiagen, Hilden, Germany) according to manufacturer’s instructions. Polymerase chain reaction (PCR) and genotyping procedures for the α-thalassemia, 3.7 kb deletion was done as previously described [21], with one forward primer 3.7F (5′-AAGTCCACCCCTTCCTTCCTCACC-3′) and two reverse primers, 3.7R1 (5′-ATGAGAGAAATGTTCTGGCACCTGCACTTG-3′) and 3.7R2 (5′-ATCCCCTCCTCCCGCCCCTGCCTTTTC-3′). PCR products with 2,213 bp were assigned as no α-thalassemia and those with 1,963 bp as homozygous α^+^ AT. Products with 2,213 bp and 1963 bp were assigned as heterozygous α^+^ AT. The PCR and genotyping characterization of G6PD deficiency was done as previously described [22], using forward primer (5′-CTGGCCAAGAAGAAGATCTACCC-3′) and reverse primer (5′-GAGAAAACGCAGCAGAGCACAG 3′). The restriction fragment length polymorphism (RFLP) products with 300 bp and 180 bp were assigned as normal, 180 bp and 120 bp were assigned as homozygotes female/hemizygous male, and 300 bp, 180 bp, and 120 bp were termed as heterozygotes. Hemoglobin sub typing for SCT/SCD was performed by PCR RFLP and gel electrophoresis as explained by Modiano [23]. In the first round of RFLP with *MnI*I reactions, products with 173 bp, 109 bp, and 60 bp were assigned as HbAA, ambiguous products with 173 bp, 109 bp, and 76 bp were assigned as HbCC/SS/SC and those with 173 bp, 109 bp, 76bp, and 60 bp were assigned as HbAC/AS. In a second round of RFLP with *Dde*I reaction to resolve ambiguities from the first round, products with 331 bp were assigned as HbSS, and those with 130 bp, 201 bp, and 331 bp were assigned as HbAS.

### Sample size and power considerations

The study was exploratory and had funds to obtain hemoglobinopathy information only on a sub-set of CICADA participants. In the entire CICADA cohort, diabetes was diagnosed using HbA1c in 13% of participants, and an additional 17% were pre-diabetic [19]. Assuming a higher prevalence of PD/DM among participants without hemoglobinopathies of 32%, with 80% statistical power, the study could detect PD/DM among participants with hemoglobinopathies of 19%, with at least 80% power.

### Data analysis

Data were analysed in STATA version 13 (StataCorp, College Station, Texas, USA). Descriptive analysis was done using histograms for shape distributions. Differences between parametric continuous variables were compared using t-test and one-way ANOVA, while non-parametric variables were compared using Kruskal Wallis test. Chi-square tests were used to compare categorical variables. The primary outcomes were PD/DM by (1) HbA1c and (2) OGTT. The main exposures were (1) SCT/SCD, (2) α-thalassemia and (3) G6PD deficiency. Logistic regression was used to examine associations of these exposures with PD/DM diagnosis by HbA1c and OGTT. Factors identified with p < 0.2 in univariable analysis were included in multivariable models.

For each hemoglobinopathy the comparison group comprised participants without that specific trait, even though they may have had one or both of the other traits investigated. Other factors considered in models were categorized age, sex, BMI, socioeconomic status, HIV status, alcohol use, smoking status, and hemoglobin level.

Non-parametric receiver operating characteristic (ROC) analysis was used to calculate the area under the ROC curves (AUROC), sensitivity and specificity. The calculated AUROC, sensitivity and specificity were used to assess the discriminative ability of HbA1c to identify the combined presence of PD/DM defined by the gold standard 2-hour OGTT. We present results stratified by SCT status.

Because there is evidence that both HIV infection and ART are associated with diabetes [24], interactions between HIV status and SCT, α-thalassemia, and G6PD deficiency in relation to PD/DM diagnosis by both HbA1c and OGTT were explored.

Sensitivity analysis was also considered for the final multivariable models using two methods: logistic regression models where those with other hemoglobinopathies were removed from the comparison group and multinomial regression with no diabetes as the reference and compared separately to the prediabetes and diabetes groups.

## Results

### Participant characteristics and hemoglobin variants

**Fig 1:**
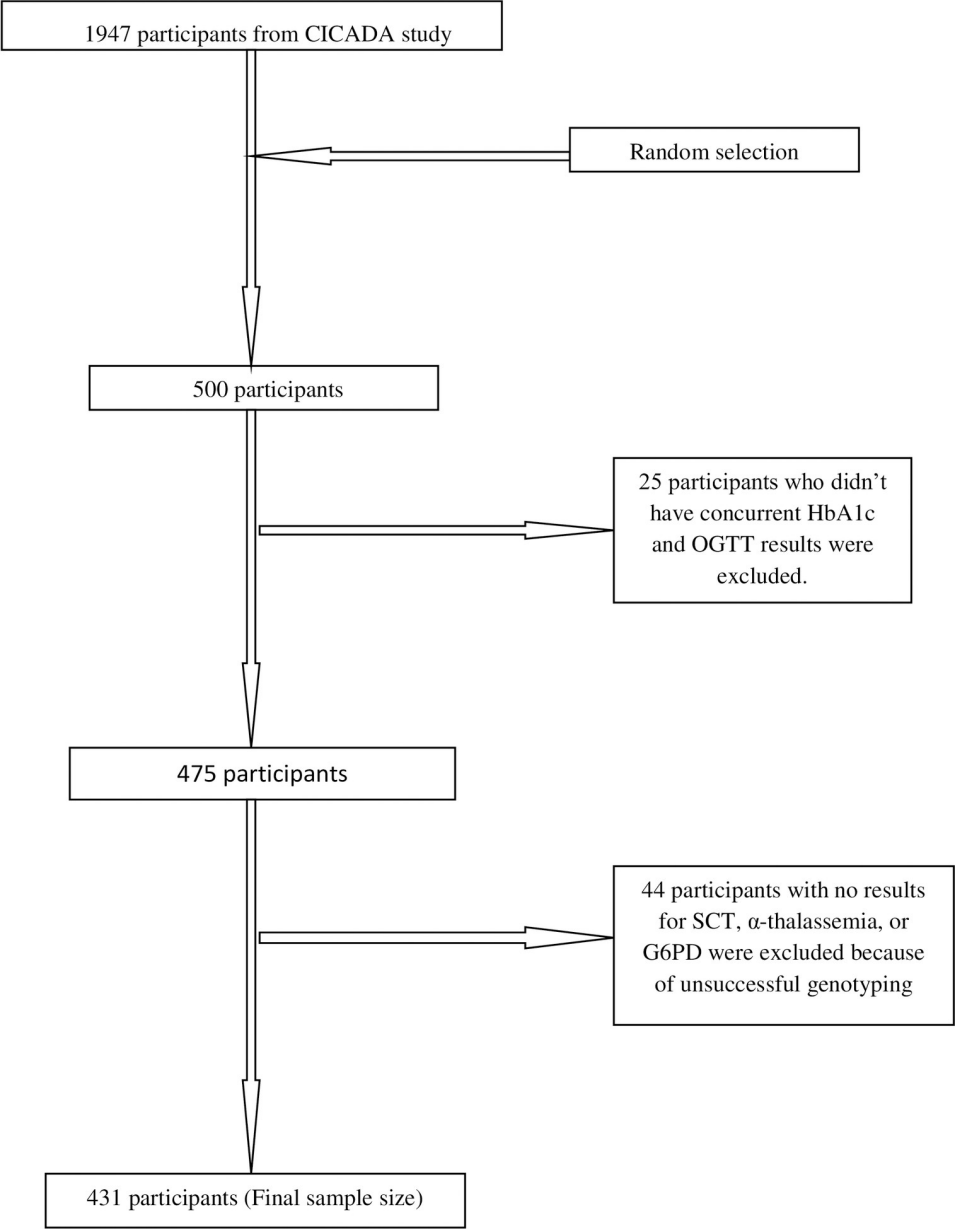
Participant enrollment and inclusion in analysis.

CICADA recruited 1947 participants in 2016–2017; among them, PD/DM was diagnosed in 582/1944 (29.9%) by HbA1c and in 972/1941 (50.1%) by OGTT. We randomly selected 500/1947 (26%) participants from the total CICADA cohort for inclusion in this sub-study. After excluding 25/500 (5.0%) samples that lacked concurrent HbA1c and 2-hour OGTT measurements and 44/500 (8.8%) samples with unsuccessful genotyping due to poor quality/quantity of extracted DNA or poor visualisation of DNA bands on agarose gel (Fig 1), we analysed 431/500 (86.2%) of the randomly selected participants.

The mean age of included participants was 40.5 (SD11.6) years; 261/431 (61%) were female. Most participants, 306/431 (71%) were living with HIV: 79/306 (26%) were established on ART and 227/306 (74%) not yet on ART (Table 1). Characteristics of sub-study participants were similar to those not included (S1 Table). SCT (HbAS) was found in 110/431 (25.5%) participants; no participants had SCD. Heterozygous and homozygous α^+^ AT were found in 186/431 (43.1%) and 52/431 (12.1%), respectively (Table 1). G6PD deficiency was the least common of the traits investigated: 40/431 (9.3%) were female heterozygotes (G6PD(A)), while 24/431 (5.6%) males and 4/431 (0.9%) females were hemizygotes and homozygotes (G6PD(A-)), respectively.

**Table 1:** Background characteristics of the study population.

Characteristic	n = 431
**Age,** mean (SD), years	40.5 (11.6)
**Female,** n (%)	261 (60.6)
**Socioeconomic terciles,** n (%)	
Low	129 (30%)
Middle	142 (33%)
Upper	159 (37%)
**Body mass index,** mean (SD), (kg/m^2^)	21.9 (4.5)
**Hemoglobin,** mean (SD), (g/dl)	12.3 (2.4)
**HIV status,** n (%)	
HIV-negative	125 (29.0)
HIV-positive not on ART	227 (52.7)
HIV-positive on ART	79 (18.3)
**Sickle cell**, n (%)	
Normal (HbAA)	321 (74.5)
Sickle cell trait (HbAS)	110 (25.5)
Sickle cell disease (HbSS)	0 (0.0)
**α-thalassemia**, n (%)	
No thalassemia	193 (44.8)
Homozygousα^+^ AT	52 (12.1)
Heterozygous α^+^ AT	186 (43.1)
**G6PD deficiency**, n (%)	
Normal G6PD (G6PD(B))	263 (84.2)
Hemizygous (G6PD(A-))	24 (5.6)
Homozygous (G6PD(A-)	4 (0.9)
Heterozygous (G6PD(A))	40 (9.3)
**HbA1c level,** mean (SD), (%)	5.7 (1.1)
**2-hr glucose level in OGTT** mean (SD), (mmol/L)	8.2 (2.4)

HIV, Human Immunodeficiency Virus; ART, antiretroviral therapy; G6PD, glucose-6-phosphate dehydrogenase; HbA1c, Hemoglobin A1c; OGTT, oral glucose tolerance test.

The most prevalent combinations were SCT plus heterozygous α^+^ AT without G6PD deficiency (HbAS, heterozygous α^+^ AT, G6PD(B)) in 41/431 (10.2%), followed by heterozygous α^+^ AT plus heterozygous G6PD deficiency without SCT (HbAA, heterozygous α^+^ AT, G6PD(A)) in 18/431 (4.2%) and SCT plus homozygous α^+^ AT without G6PD deficiency (HbAS, homozygous α^+^ AT, G6PD(B)) in 11/431 (2.6%) (Table 2).

**Table 2:** Hemoglobin and G6PD deficiency subtypes of the study population (n = 431)^1^.

Red blood cell polymorphisms	Alleles	N (%)
No hemoglobinopathy	HbAA, no α-thalassemia, G6PD(B)	127 (29.5)
Sickle cell trait (SCT)	HbAS, no α-thalassemia, G6PD(B)	38 (8.8)
Homozygous α^+^ AT	HbAA, homozygous α^+^ AT, G6PD(B)	33 (7.7)
Heterozygous α^+^ AT	HbAA, heterozygous α^+^ AT, G6PD(B)	110 (25.5)
Homozygous/hemizygous G6PD deficiency^2^	HbAA, no α-thalassemia, G6PD(A-)	11 (2.6)
SCT, homozygous α^+^ AT, heterozygous G6PD deficiency	HbAA, homozygous α^+^ AT, G6PD(A)	01 (0.2)
Heterozygous G6PD deficiency	HbAA, no α-thalassemia, G6PD(A)	06 (1.4)
SCT, heterozygous α^+^ AT, heterozygous G6PD deficiency	HbAS, heterozygous α^+^ AT, G6PD(A)	05 (1.2)
SCT, no α-thalassemia, homozygous G6PD deficiency	HbAS, no α-thalassemia, G6PD(A-)	04 (0.9)
SCT, homozygous α^+^ AT, no G6PD deficiency	HbAS, homozygous α^+^ AT, G6PD(B)	11 (2.6)
SCT, no α-thalassemia, heterozygous G6PD deficiency	HbAS, no α-thalassemia, G6PD(A)	07 (1.6)
SCT, heterozygous α^+^ AT, no G6PD deficiency	HbAS, heterozygous α^+^AT, G6PD(B)	44 (10.2)
No SCT, homozygous α^+^ AT, heterozygous G6PD deficiency	HbAA, homozygous α^+^ AT, G6PD(A)	03 (0.7)
No SCT, heterozygous α^+^ AT, homozygous G6PD deficiency	HbAA, heterozygous α^+^ AT, G6PD(A-)	09 (2.1)
No SCT, heterozygous α^+^ AT, heterozygous G6PD deficiency	HbAA, heterozygous α^+^ AT, G6PD(A)	18 (4.0)
No SCT, homozygous α^+^ AT, homozygous G6PD deficiency	HbAA, homozygous α^+^ AT, G6PD(A-)	04 (1.0)

G6PD; glucose-6-phosphate-dehydrogenase.

^1^Data are number (%).

^2^Homozygous is for females while hemizygous is for males.

Overall, 92/431 (21.4%) participants were diagnosed with PD/DM by HbA1c only; among them, 9/92 (9.8%) had SCT. By OGTT only, 103/431 (23.9%) had PD/DM and 47/103 (45.6%) of these had SCT. By both HbA1c and OGTT, 108/431 (25.1%) participants were diagnosed with PD/DM; of these 13/108 (12.0%) had SCT.

### PD/DM diagnosis by HbA1c

**Fig 2:**
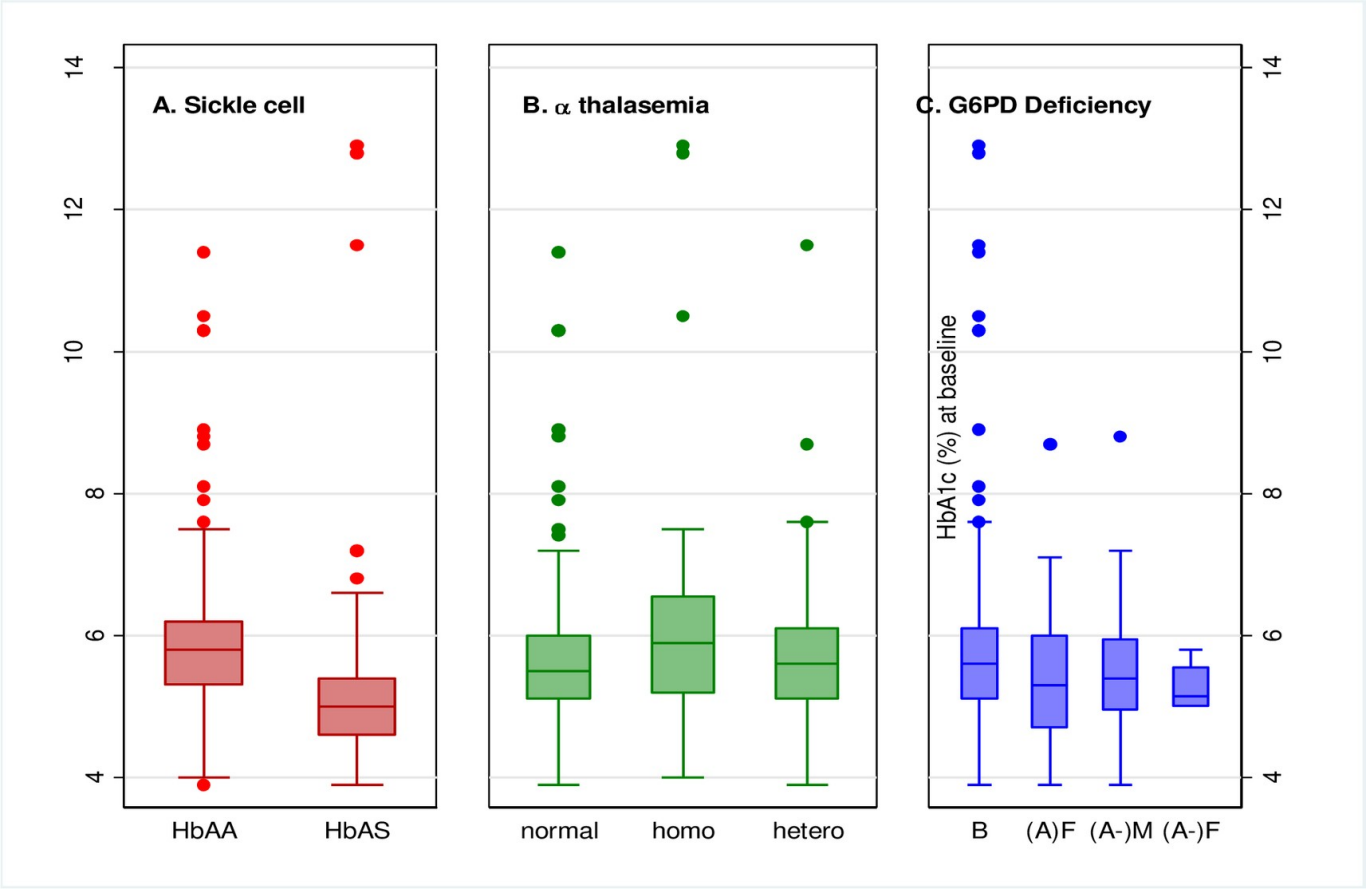
**A: Boxplots of HbA1c comparing sickle cell trait status.** By using HbA1c, 29/52 (55.8%) of participants with homozygous α^+^ AT and 88/186 (47.3%) of participants with heterozygous α+ AT had PD/DM, while among those without α-thalassemia, 83/193 (43.0%) did (Table 3). α-thalassemia was not associated with diagnosis of PD/DM by HbA1c (overall p = 0.37 in the adjusted model, Table 3). However, there was a tendency for increased odds of PD/DM among those with homozygous α-thalassemia (adjusted OR 1.61, 95% CI: 0.82, 3.16) (Table 3). Median HbA1c level was highest among homozygotes (S2 Table) and there was an upward shift in the distribution of HbA1c among homozygotes compared to those without α-thalassemia (Fig 2B). **B: Boxplots of HbA1c comparing α-thalassemia status.** In participants with G6PD deficiency, 8/24 (32.3%) hemizygous males, 1/4 (25.0%) homozygous female, and 15/40 (37.5%) heterozygous females had PD/DM by HbA1c, compared to 176/363 (48.5%) of those with no G6PD deficiency (Table 3). Among people with hemizygous or homozygous G6PD deficiency, there was weak evidence of decreased odds of PD/DM diagnosis by HbA1c compared to those without G6PD deficiency (overall p = 0.06 in adjusted model Table 3). Compared to those without G6PD deficiency, there was decreased odds of PD/DM in hemizygotes (adjusted OR 0.40, 95% CI: 0.15, 1.11), homozygotes (adjusted OR 0.19, 95% CI: 0.02, 2.07) and heterozygotes (adjusted OR 0.53, 95% CI: 0.25, 1.14) (Table 3). Median HbA1c was slightly lower in hemizygous, homozygous and heterozygous G6PD deficiency (S2 Table), and their distributions were shifted down compared to those without G6PD deficiency (Fig 2C). **C: Boxplots of HbA1c comparing G6PD deficiency status**. There was no evidence for interaction between HIV status and SCT (p = 0.62), homozygous or heterozygous α+ AT (p = 0.49), or G6PD deficiency (p = 0.40) with regard to PD/DM diagnosis by HbA1c. Sensitivity analyses yielded results that were materially similar to those presented here.

Fewer participants with SCT, 22/110 (20.0%), were diagnosed with PD/DM by HbA1c compared to those without SCT, 178/321 (55.5%). There was strong evidence that among participants with SCT, there was reduced odds of PD/DM diagnosis by HbA1c in both unadjusted and adjusted analyses (adjusted OR 0.15, 95% CI: 0.08, 0.26) (Table 3). Median HbA1c level among SCT participants was (5.8% (5.3:6.2) compared to (5.6% (5.1:6.1)) of those without SCT (S2 Table). The distribution of HbA1c results was shifted downward among those with SCT compared to those without (Fig 2A).

**Table 3:** Association of hemoglobinopathies and G6PD deficiency with prediabetes/diabetes diagnosis by HbA1c and oral glucose tolerance test (OGTT).

PD/DM by HbA1c							
Characteristic	PD/DM Prevalence, n (%)	Crude OR (95% CI)^1^	P^1^	Overall P^1^	Adjusted OR (95% CI)^2^	P^2^	Overall P^2^
**Sickle cell trait**							
No SCT^3^	178 (55.5)	**Reference**			**Reference**		
SCT	22 (20.0)	0.20 (0.12, 0.34)		< 0.001	0.15 (0.08, 0.26)		< 0.001
**α-thalassemia**							
No α-thalassemia^3^	83 (43.1)	**Reference**			**Reference**		
Homozygous α^+^ AT	29 (55.8)	1.67 (0.90, 3.10)	0.10	0.25	1.61 (0.82, 3.16)	0.17	0.37
Heterozygous α^+^ AT	88 (47.3)	1.19 (0.79, 1.78)	0.84		1.17 (0.75, 1.81)	1.81	
**G6PD deficiency**							
No G6PD deficiency^3^ (G6PD (B))	176 (48.5)	**Reference**			**Reference**		
Hemizygous (G6PD (A-))	8 (33.3)	0.53 (0.22, 1.27)	0.16	0.24	0.40 (0.15, 1.11)	0.08	0.06
Homozygous (G6PD (A-))	1 (25.0)	0.35 (0.04, 3.44)	0.37		0.19 (0.02, 2.07)	0.17	
Heterozygous (G6PD (A))	15 (37.5)	0.64 (0.33, 1.25)	0.19		0.53 (0.25, 1.14)	0.10	
**PD/DM by 2-hr OGTT**							
**Sickle cell trait**							
No SCT^3^	151 (47.0)	**Reference**			**Reference**		
SCT	60 (54.5)	1.35 (0.87, 2.09)		0.18	1.28 (0.82, 2.00)		0.28
**α-thalassemia**							
No α-thalassemia^3^	89 (46.1)	**Reference**			**Reference**		
Homozygous α^+^ AT	32 (61.5)	1.87 (1.00, 3.50)	0.05	0.14	2.00 (1.04, 3.84)	0.04	0.11
Heterozygous α^+^ AT	90 (48.4)	1.10 (0.73, 1.64)	0.66		1.15 (0.76, 1.74)	0.51	
**G6PD deficiency**							
No G6PD deficiency^3^ (G6PD (B))	181 (49.9)	**Reference**			**Reference**		
Hemizygous (G6PD (A-))	16 (66.7)	2.01 (0.84, 4.82)	0.12	0.04	1.81 (0.72, 4.59)	0.21	0.13
Homozygous (G6PD (A-))	2 (50.0)	1.01 (0.14, 7.21)	1.00		1.26 (1.16, 9.61)	0.83	
Heterozygous (G6PD (A))	12 (30.0)	0.43 (0.21, 0.87)	0.02		0.47 (0.22, 0.99)	0.05	

SCT, sickle cell trait; G6PD, glucose-6-phosphate dehydrogenase; PD/DM, prediabetes/diabetes.

^1^Unadjusted analysis; variables involved in unadjusted analysis were age, sex, socioeconomic status, smoking status, alcohol use, body mass index, HIV status and hemoglobin level.

^2^Adjusted for all variables associated with PD/DM by HbA1c or OGTT, with overall p. value of < 0.2 before adjusted. Those variables were age, sex, body mass index, HIV status and hemoglobin level.

^3^For each trait, the reference group comprised participants without that specific trait, even though they may have one or both of the other traits investigated.

### PD/DM diagnosis by OGTT

**Fig 3:**
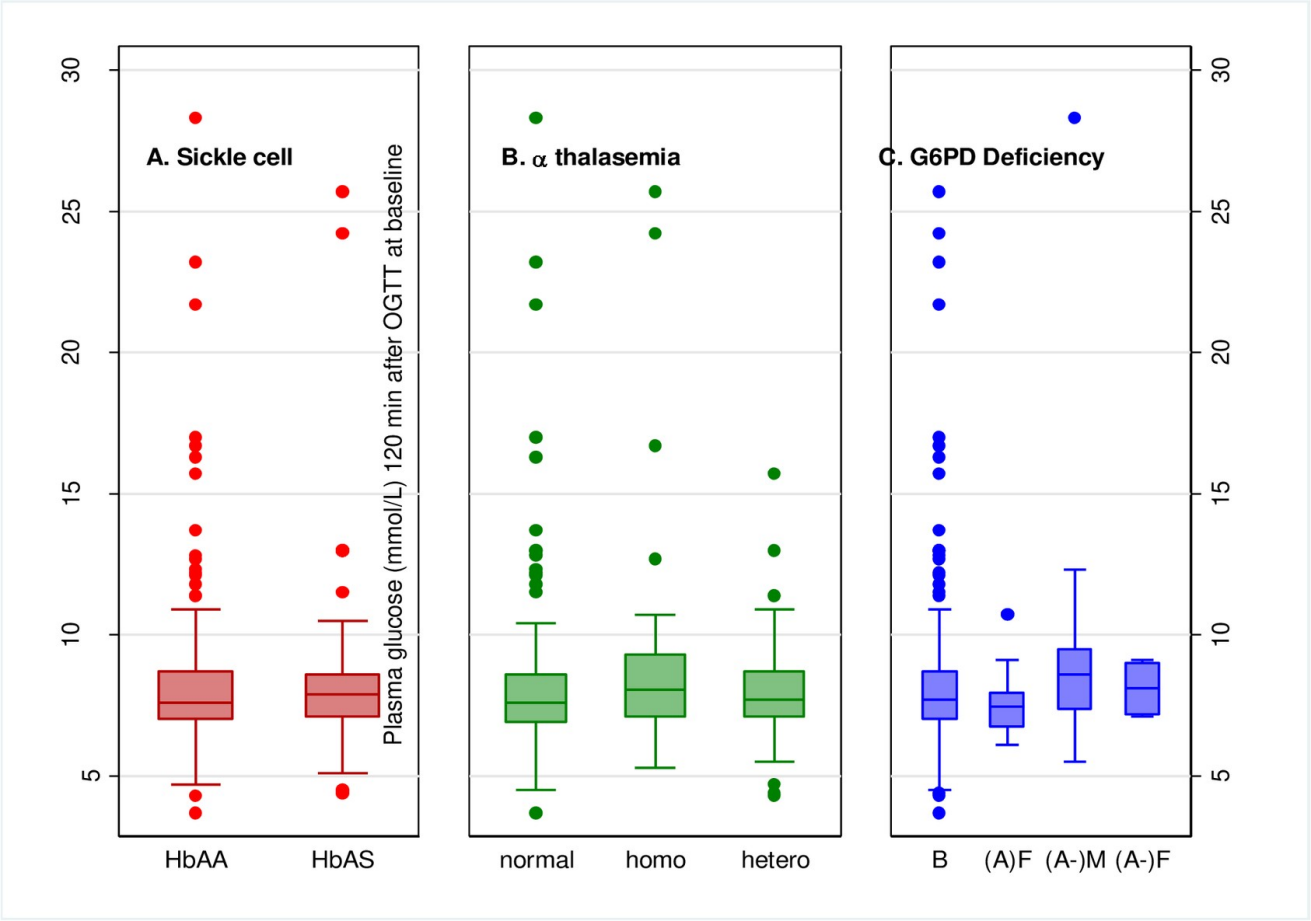
**A: Boxplots of OGTT comparing sickle cell trait status.** By OGTT, PD/DM was prevalent in 32/52 (61.5%) of participants with homozygous α^+^ AT, 90/186 (48.4%) of those with heterozygous α^+^ AT, and 89/193 (46.1%) of those without α-thalassemia (Table 3). Compared to those without α-thalassemia, participants with homozygous α^+^ AT had increased odds of being diagnosed with PD/DM by OGTT (adjusted OR 2.00, 95% CI: 1.04, 3.84) (Table 3). However, there was overall no evidence that α-thalassemia was associated with diabetes diagnosis by OGTT (overall p = 0.11 in adjusted analysis). Compared to those without α-thalassemia, the median OGTT glucose was highest among homozygotes (S2 Table) and the distributions of OGTT results were shifted upward among those with homozygous α+ AT (Fig 3B). **B: Boxplots of OGTT comparing α-thalassemia status.** Those with hemizygous, homozygous or heterozygous G6PD deficiency had PD/DM prevalence of 16/24 (66.7%), 2/4 (50.0%), and 12/40 (30.0%) respectively, while in those with normal G6PD activity, PD/DM prevalence was 181/363 (49.9%) (Table 3). Compared to those without G6PD deficiency, heterozygous females had decreased odds of PD/DM diagnosis (adjusted OR 0.47, 95% CI: 0.22, 0.99, Table 3). Median OGTT glucose was highest among hemizygotes (8.6 (7.4:9.6) mmol/L) and lowest among heterozygotes (7.5 (6.8:8.0) mmol/L) when compared to those without G6PD deficiency (7.7 (7.0:8.7) mmol/L) (S2 Table). The distribution was shifted upward among hemizygotes while in heterozygotes the distribution was shifted downward compared to those not deficient (Fig 3C). **C: Boxplots of OGTT comparing G6PD deficiency status.** No interaction was observed between HIV status and SCT (p = 0.61), α-thalassemia (p = 0.80), or G6PD deficiency (p = 0.35) with regard to PD/DM diagnosis by OGTT. Sensitivity analyses were consistent with the presented results.

The prevalence of PD/DM measured by OGTT in participants with SCT was 60/110 (54.5%) compared to 151/321 (47.0%) among those without SCT. In contrast to the findings for HbA1c, there was no evidence that SCT was associated with PD/DM diagnosis by OGTT (adjusted OR 1.28, 95% CI: 0.82, 2.00) compared to those without SCT (Table 3). There was also no evidence of a difference in the distributions of OGTT results comparing those with and without SCT (Fig 3A).

### Discriminative ability of HbA1c to Identify PD/DM

**Fig 4:**
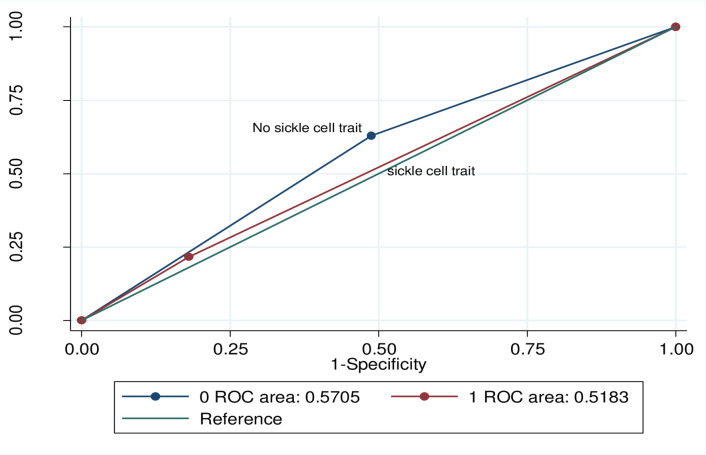
Comparison of discriminative ability of HbA1c to identify PD/DM by sickle cell trait status using 2-hour oral glucose tolerance test.

Discriminative ability of HbA1c to identify PD/DM was non-significantly lower among participants with SCT (AUROC, 0.52; 95% CI, 0.44, 0.59) vs. without SCT (AUROC, 0.57; 95% CI, 0.52, 0.62) when using OGTT-defined PD/DM (p = 0.27, Fig 4). In comparison to the OGTT, among those with SCT the sensitivity of HbA1c was 21.7% (95% CI: (12.1, 34.2) and specificity was 82.0% (95% CI: 68.6, 91.4), while among those without SCT, sensitivity was 62.9% (95% CI: 54.3, 70.2) and specificity was 51.2% (95% CI: 43.4, 58.9) for the diagnosis of PD/DM.

## Discussion

In this cross-sectional study among Tanzanian adults with and without HIV, we assessed the utility of HbA1c for diagnosing diabetes or prediabetes in people with SCT, α-thalassemia, and G6PD deficiency. SCT was strongly associated with lower values of HbA1c and therefore a lower prevalence estimate of PD/DM compared to those without SCT. This was not the case when using OGTT. HbA1c also appeared to underestimate PD/DM in participants with hemizygous or homozygous G6PD deficiency compared to participants without G6PD deficiency.

Our findings of correlation between SCT and HbA1c are only applicable to the HemoCue HbA1c 501 device and methods used here, as they contrast with other studies’ findings that used different methods and observed no correlation between SCT and HbA1c. The studies of Sumner *et al* and Bleyer *et al* were conducted in populations with high prevalences of SCT, i.e., 28% and 21% [6,8], but they combined two different hemoglobin types, i.e., HbAS and HbC traits, as one group and used different methods for measuring glycated HbA1c which may explain the different results. They conducted the analyses with cation exchange column chromatography on automated high performance liquid chromatography (HPLC) (VariantIITurbo, BioRad Laboratories, Hercules California USA) [6,8], with further confirmation by Boronate Affinity Chromatography on Premier Hb9210 analyser (Trinity Biotech, Bray, Ireland) [6].

Using OGTT, we observed that participants with either homozygous α^+^ AT or hemizygous G6PD deficiency trended toward higher prevalence of PD/DM diagnosis compared to those with no α-thalassemia or G6PD deficiency. The association was not strong and the mechanism behind it is not well understood, but this finding was also reported in other studies [25,26]. To further investigate the associations, studies with larger sample sizes should be conducted. To elucidate mechanisms, clinical and translational research such as glucose/insulin kinetics studies in people with hemoglobinopathies and G6PD deficiency is required.

Our study has a number of strengths. First, samples were randomly selected from stored blood samples of a large cohort study, CICADA, conducted in Mwanza, northwestern Tanzania, commonly known to have a high prevalence of hemoglobinopathies, especially SCT and α-thalassemia. This enabled us to assess the hypothesized associations within a modest sample size. Second, to our knowledge there are very few studies that have examined associations of HbA1c with SCT, α-thalassemia and G6PD deficiency in a single cohort. Many studies have established an association of one trait, e.g., SCT, with HbA1c, without considering other traits that impact red blood cells.

The study also had limitations. First, we included only Tanzanians and found the only abnormal hemoglobin type to be HbAS. Hemoglobin variants other than HbAS, for which we did not test, could also potentially affect the validity of HbA1c measurements [4,27]. HbAS is not the only trait of concern, therefore studies that involve populations of more than one country and different abnormal hemoglobin variants, e.g., HbAC, HbCC, HbSC and HbE are needed to confirm and complement our findings. Second, validation of these findings with other HbA1c assays could help to explain the disparate PD/DM prevalence by HbA1c reported by other investigators in people with SCT, α-thalassemia or G6PD deficiency.

## Conclusions

Our findings add to the body of scientific evidence that hemoglobinopathies can influence diagnosis of PD/DM using HbA1c. Even though our findings relied on a single device, HemoCue HbA1c 501, we suggest that HbA1c be used and interpreted with caution in areas with high prevalences of hemoglobinopathies such as sub-Saharan Africa. Economical and cost-effective screening strategies for hemoglobinopathies and diabetes case management guidelines, e.g., instrument-specific glycated HbA1c thresholds for SCT individuals should be considered. When using OGTT, higher prevalence of PD/DM was recorded among participants with homozygous α-thalassemia and hemizygous G6PD deficiency cohorts; further larger scale studies are warranted to investigate these associations.

## Supporting information

S1 Table Comparison of full cohort characteristics with sub-study. (DOCX)

S2 Table Background characteristics by sickle cell trait, α-thalassemia and G6PD deficiency. (DOCX)

## References

[1] Shaw JE, Sicree RA, Zimmet PZ. Global estimates of the prevalence of diabetes for 2010 and 2030. Diabetes research and clinical practice. 2010;87(1):4–14. 10.1016/j.diabres.2009.10.007 19896746

[2] Organization WH. Definition and diagnosis of diabetes mellitus and intermediate hyperglycaemia: report of a WHO/IDF consultation. 2006.

[3] WHO Guidelines Approved by the Guidelines Review Committee. Use of Glycated Haemoglobin (HbA1c) in the Diagnosis of Diabetes Mellitus: Abbreviated Report of a WHO Consultation. Geneva: World Health Organization Copyright (c) World Health Organization 2011; 2011. 26158184

[4] Begum A, Muttalib MA, Arefin MN, Hoque MR, Sheme ZA, Akter N, et al Challenges in HbA1C Level as a Diagnostic Tool of Diabetes and Pre-Diabetes in Middle-Aged Population: The Bangladesh Study. Mymensingh medical journal: MMJ. 2016;25(4):721–5. 27941737

[5] McCurdy PR. 32-DFP and 51-Cr for measurement of red cell life span in abnormal hemoglobin syndromes. Blood. 1969;33(2):214–24. 5766311

[6] Sumner AE, Thoreson CK, O'Connor MY, Ricks M, Chung ST, Tulloch-Reid MK, et al Detection of abnormal glucose tolerance in Africans is improved by combining A1C with fasting glucose: the Africans in America Study. Diabetes care. 2015;38(2):213–9. 10.2337/dc14-1179 25338926 PMC4302255

[7] Suarez RM, Buso R, Meyer LM, Olavarrieta ST. Distribution of abnormal hemoglobins in Puerto Rico and survival studies of red blood cells using Cr51. Blood. 1959;14(3):255–61. 13628823

[8] Bleyer AJ, Vidya S, Sujata L, Russell GB, Akinnifesi D, Hire D, et al The impact of sickle cell trait on glycated haemoglobin in diabetes mellitus. Diabetic medicine: a journal of the British Diabetic Association. 2010;27(9):1012–6. 10.1111/j.1464-5491.2010.03050.x 20722674

[9] Lacy ME, Wellenius GA, Sumner AE, Correa A, Carnethon MR, Liem RI, et al Association of Sickle Cell Trait With Hemoglobin A1c in African Americans. Jama. 2017;317(5):507–15. 10.1001/jama.2016.21035 28170479 PMC5713881

[10] Muncie HL Jr., Campbell J. Alpha and beta thalassemia. American family physician. 2009;80(4):339–44. 19678601

[11] Al-Fadhli SM, Ahmad AA, Al-Jafar HA. Effect of sickle cell trait and B-Thalassemia minor on determinations of HbA1c by an immunoassay method. Saudi medical journal. 2001;22(8):686–9. 11573113

[12] Mwakasungula S, Schindler T, Jongo S, Moreno E, Kamaka K, Mohammed M, et al Red blood cell indices and prevalence of hemoglobinopathies and glucose 6 phosphate dehydrogenase deficiencies in male Tanzanian residents of Dar es Salaam. International journal of molecular epidemiology and genetics. 2014;5(4):185–94. 25755846 PMC4348704

[13] Ambrose EE, Makani J, Chami N, Masoza T, Kabyemera R, Peck RN, et al High birth prevalence of sickle cell disease in Northwestern Tanzania. Pediatric blood & cancer. 2018;65(1). 10.1002/pbc.26735 28766840 PMC5701733

[14] Badr M, Afifi RAA. Pattern of Hemolytic Anemia Among Egyptian Pediatric Emergency Department Patients. Pediatric emergency care. 2020;36(3):153–7. 10.1097/PEC.0000000000002053 32108744

[15] Wheeler E, Leong A, Liu CT. Impact of common genetic determinants of Hemoglobin A1c on type 2 diabetes risk and diagnosis in ancestrally diverse populations: A transethnic genome-wide meta-analysis. 2017;14(9):e1002383 10.1371/journal.pmed.1002383 28898252 PMC5595282

[16] Leong A, Lim VJY, Wang C, Chai JF, Dorajoo R, Heng CK, et al Association of G6PD variants with hemoglobin A1c and impact on diabetes diagnosis in East Asian individuals. 2020;8(1). 10.1136/bmjdrc-2019-001091 32209585 PMC7103857

[17] Filmer D, Pritchett LH. Estimating wealth effects without expenditure data—or tears: an application to educational enrollments in states of India. Demography. 2001;38(1):115–32. 10.1353/dem.2001.0003 11227840

[18] Wollner M, Paulo Roberto B-B, Alysson Roncally SC, Jurandir N, Edil LS. Accuracy of the WHO's body mass index cut-off points to measure gender- and age-specific obesity in middle-aged adults living in the city of Rio de Janeiro, Brazil. J Public Health Res. 2017;6(2):904 10.4081/jphr.2017.904 29071256 PMC5641638

[19] Jeremiah K, Filteau S, Faurholt-Jepsen D. Diabetes prevalence by HbA1c and oral glucose tolerance test among HIV-infected and uninfected Tanzanian adults. 2020;15(4):e0230723 10.1371/journal.pone.0230723 32267855 PMC7141607

[20] Cappellini MD, Motta I. Anemia in Clinical Practice-Definition and Classification: Does Hemoglobin Change With Aging? Seminars in hematology. 2015;52(4):261–9. 10.1053/j.seminhematol.2015.07.006 26404438

[21] Liu YT, Old JM, Miles K, Fisher CA, Weatherall DJ, Clegg JB. Rapid detection of alpha-thalassaemia deletions and alpha-globin gene triplication by multiplex polymerase chain reactions. British journal of haematology. 2000;108(2):295–9. 10.1046/j.1365-2141.2000.01870.x 10691858

[22] Fanello CI, Karema C, Avellino P, Bancone G, Uwimana A, Lee SJ, et al High risk of severe anaemia after chlorproguanil-dapsone+artesunate antimalarial treatment in patients with G6PD (A-) deficiency. PloS one. 2008;3(12):e4031 10.1371/journal.pone.0004031 19112496 PMC2603295

[23] Modiano D, Luoni G, Sirima BS, Simpore J, Verra F, Konate A, et al Haemoglobin C protects against clinical Plasmodium falciparum malaria. Nature. 2001;414(6861):305–8. 10.1038/35104556 11713529

[24] Noubissi EC, Katte JC, Sobngwi E. Diabetes and HIV. Current diabetes reports. 2018;18(11):125 10.1007/s11892-018-1076-3 30294763

[25] Lai YK, Lai NM, Lee SW. Glucose-6-phosphate dehydrogenase deficiency and risk of diabetes: a systematic review and meta-analysis. Annals of hematology. 2017;96(5):839–45. 10.1007/s00277-017-2945-6 28197721

[26] Lao TT, Ho LF. alpha-Thalassaemia trait and gestational diabetes mellitus in Hong Kong. Diabetologia. 2001;44(8):966–71. 10.1007/s001250100594 11484072

[27] Sultana TA, Sheme ZA, Sultana GS, Sultana B, Mishu FA, Khan NZ, et al Challenges in HbA1c Analysis and Reporting in Patients with Variant Hemoglobins. Mymensingh medical journal: MMJ. 2016;25(2):248–54. 27277356

