# Supplementary material for: Influence of hemoglobinopathies and glucose-6-phosphate dehydrogenase deficiency on diagnosis of diabetes by HbA1c among Tanzanian adults with and without HIV: A cross-sectional study
Source: PLoS One. 2020 Dec 31;15(12):e0244782. doi: 10.1371/journal.pone.0244782 (PMC7775052; doi:10.1371/journal.pone.0244782)
Supplement: S1 Table — (DOCX) [file pone.0244782.s001.docx]

| **S1 Table: Comparison of full cohort characteristics with sub-study** | | | | |
| --- | --- | --- | --- | --- |
| **Characteristics** | **Overall**  **(N=1947)^1^** | **Not in sub-study (n=1516),**  **mean (SD)**  **or n (%)** | **In sub-study**  **(n=431),**  **mean (SD)**  **or n (%)** | **P** |
| **Age (years)** | 1947 | 1.4 (1.0) | 1.4 (1.0) | 0.30 |
| 18-30 |  | 327 (21.6) | 93 (21.6) | 0.19 |
| 31-40 |  | 469 (30.9) | 154 (35.7) |  |
| 41-50 |  | 420 (27.7) | 101 (23.4) |  |
| >50 |  | 300 (19.8) | 83 (19.3) |  |
| **Sex** | 1947 |  |  |  |
| Females |  | 896 (59.1) | 261 (60.6) | 0.59 |
| Male |  | 620 (40.9) | 170 (39.4) |  |
| **Socioeconomic status** |  |  |  |  |
| Low | 1942 | 519 (34.3) | 129 (30.0) | 0.13 |
| Middle |  | 505 (33.4) | 142 (33.0) |  |
| Upper |  | 488 (32.3) | 159 (37.0) |  |
| **Smoking status** | 1942 |  |  |  |
| Never smoked |  | 1147 (75.9) | 327 (76.1) | 0.65 |
| Past smoked |  | 219 (14.5) | 67 (15.6) |  |
| Current smoking |  | 146 (9.6) | 36 (8.3) |  |
| **Alcohol use** | 1942 |  |  |  |
| No |  | 447 (29.6) | 106 (24.7) | 0.05 |
| Yes |  | 1065 (70.4) | 324 (75.3) |  |
| **Body mass index (kg/m^2^)** | 1946 | 21.8 (4.5) | 22.2 (4.6) | 0.14 |
| Underweight/Normal |  | 1220 (80.5) | 336 (78.0) | 0.24 |
| Overweight/Obesity |  | 295 (19.5) | 95 (22.0) |  |
| **HIV status** | 1947 |  |  |  |
| HIV-negative |  | 530 (35.0) | 125 (29.0) | 0.07 |
| HIV-positive not on ART |  | 729 (48.0) | 227 (52.7) |  |
| HIV-positive on ART |  | 257 (17.0) | 79 (18.3) |  |
| **Hemoglobin(g/dl)** | 1947 | 12.3 (2.4) | 12·3 (2.4) | 0.75 |
| No anemia |  | 862 (56.9) | 256 (59.4) | 0.36 |
| Anemia |  | 652 (43.1) | 175 (40.6) |  |
| **HbA1c level, (%)** | 1944 | 5.7 (1.0) | 5.7 (1.1) | 0.81 |
| No PD/DM |  | 836 (55.2) | 231 (53.6) | 0.54 |
| PD/DM |  | 677 (44.8) | 200 (46.4) |  |
| **2-hr-glucose-level in OGTT, (mmol/L)** | 1941 | 8.2 (2.4) | 8.1 (2.4) | 0.36 |
| No PD/DM |  | 749 (49.6) | 220 (51.0) | 0.59 |
| PD/DM |  | 761 (50.4) | 211 (49.0) |  |
| HbA1c, hemoglobin A1c; PD/DM, prediabetes/diabetes.  ^1^Some variables do not sum to 1947 due to missing values. | | | | |
