# Supplementary material for: Influence of hemoglobinopathies and glucose-6-phosphate dehydrogenase deficiency on diagnosis of diabetes by HbA1c among Tanzanian adults with and without HIV: A cross-sectional study
Source: PLoS One. 2020 Dec 31;15(12):e0244782. doi: 10.1371/journal.pone.0244782 (PMC7775052; doi:10.1371/journal.pone.0244782)
Supplement: S2 Table — (DOCX) [file pone.0244782.s002.docx]

| **S2 Table: Background characteristics by sickle cell trait, α-thalassemia and G6PD deficiency^1^** | | | | | | | | | | | | |
| --- | --- | --- | --- | --- | --- | --- | --- | --- | --- | --- | --- | --- |
| **Characteristics** | **No SCT** | **SCT** | **P** | **No**  **α-thalassemia** | **homozygous**  **α^+^ AT** | **heterozygous**  **α^+^ AT** | **P** | **No G6PD**  **deficiency** | **Female**  **G6PD(A)** | **Males**  **G6PD(A-)** | **Female**  **G6PD(A-)** | **P** |
| **Age**(years) | 40.7 (11.7) | 40.2 (11.4) | 0.71 | 40.9 (12.0) | 42.5 (11.8) | 39.6 (11.0) | 0.47 | 40.7 (11.8) | 38.4 (8.9) | 42.9 (13.0) | 32.0 (3.6) | 0.03 |
| 18-30 | 69 (21.5) | 24 (21.8) | 0.99 | 42 (21.8) | 9 (17.3) | 42 (22.6) | 0.41 | 81 (22.3) | 7 (17.5) | 7 (12.5) | 2 (50.0) | 0.30 |
| 31-40 | 114 (35.5) | 40 (36.4) |  | 64 (33.2) | 15 (28.9) | 75 (40.3) |  | 126 (34.7) | 17 (42.5) | 9 (37.5) | 2 (50.0) |  |
| 41-50 | 75 (23.4) | 26 (23.6) |  | 46 (23.8) | 16 (30.8) | 39 (21.0) |  | 81 (22.3) | 13 (32.5) | 7 (29.2) | 0 (0.0) |  |
| > 50 | 63 (19.6) | 20 (18.2) |  | 41 (21.2) | 12 (23.0) | 30 (16.1) |  | 75 (20.7) | 3 (7.5) | 5 (20.8) | 0 (0.0) |  |
| **Sex** |  |  |  |  |  |  |  |  |  |  |  |  |
| Females | 197 (61.4) | 64 (58.2) | 0.56 | 117 (60.6) | 33 (63.5) | 111 (59.7) | 0.89 | 217 (59.8) | 40 (100.0) | 0 (0.0) | 4 (100.0) | < 0.001^2^ |
| Male | 124 (38.6) | 46 (41.8) |  | 76 (39.4) | 19 (36.5) | 75 (40.3) |  | 146 (40.2) | 0 (0.0) | 24 (100.0) | 0 (0.0) |  |
| **Socioeconomic status** |  |  |  |  |  |  |  |  |  |  |  |  |
| Low | 91 (28.4) | 38 (34.6) | 0.27 | 48 (24.9) | 15 (29.4) | 66 (35.5) | 0.12 | 105 (29.0) | 13 (32.5) | 10 (41.7) | 1 (25.0) | 0.57 |
| Middle | 104 (32.5) | 38 (34.6) |  | 71 (36.8) | 20 (39.2) | 51 (27.4) |  | 122 (33.7) | 13 (32.5) | 7 (29.2) | 0 (0.0) |  |
| Upper | 125 (39.1) | 34 (30.8) |  | 74 (38.3) | 16 (31.4) | 69 (37.1) |  | 135 (37.3) | 14 ( 35.0) | 7 (29.1) | 3 (75.0) |  |
| **Body mass index** (kg/m^2^) | 22.4 (4.8) | 21.5 (4.1) | 0.09 | 22.2 (4.7) | 22.5 (4.4) | 22.3 (4.7) | 0.87 | 22.2 (4.8) | 22.9 (4.4) | 20.8 (2.9) | 22.2 (2.9) | 0.04^3^ |
| Underweight/Normal | 242 (75.4) | 94 (85.4) | 0.02^3^ | 151 (78.2) | 42 (80.8) | 143 (76.9) | 0.83 | 283 (78.0) | 29 (72.5) | 21 (87.5) | 3 (75.0) | 0.58 |
| Overweight/Obesity | 79 (24.6) | 16 (14.6) |  | 42 (21.8) | 10 (19.2) | 43 (23.1) |  | 80 (22.0) | 11 (27.5) | 3 (12.5) | 1 (25.0) |  |
| **HIV status** |  |  |  |  |  |  |  |  |  |  |  |  |
| HIV-negative | 100 (31.2) | 25 (22.7) | 0.05 | 54 (28.0) | 15 (28.9) | 56 (30.1) | 0.88 | 104 (28.7) | 14 (35.0) | 6 (25.0) | 1 (25.0) | 0.87 |
| HIV-positive not on ART | 170 (52.9) | 57 (51.8) |  | 104 (53.9) | 25 (48.1) | 18 (52.7) |  | 192 (52.9) | 18 (45.0) | 14 (58.3) | 3 (75.0) |  |
| HIV-positive on ART | 51 (15.9) | 28 (25.5) |  | 35 (18.1) | 12 (23.0) | 32 (17.2) |  | 67 (18.4) | 8 (20.0) | 4 (16.7) | 0 (0.0) |  |
| **Hemoglobin level** (g/dl) | 12.4 (2.4) | 12.3 (2.3) | 0.55 | 12.6 (2·6) | 11.8 (2.0) | 12.4 (2.1) | 0.01^4^ | 12.4 (2.3) | 11.5 (2.1) | 13.0 (2.6) | 11.4 (1.0) | 0.32 |
| No anemia | 192 (52.8) | 64 (58.2) | 0.76 | 120 (62.2) | 25 (48.1) | 111 (59.7) | 0.18 | 220 (60.6) | 20 (50.0) | 15 (62.5) | 1 (25.0) | 0.29 |
| Anemia | 129 (40.2) | 46 (41.8) |  | 73 (37.8) | 27 (51.9) | 75 (40.3) |  | 143 (39.4) | 20 (50.0) | 9 (37.5) | 3 (75.0) |  |
| **Sickle cell trait** | - | - | - |  |  |  |  |  |  |  |  |  |
| No SCT |  |  |  | 144 (74.6) | 40 (76.9) | 137 (73.7) | 0.89 | 270 (74.4) | 27 (67.5) | 20 (83.3) | 4 (100.0) | 0.34 |
| SCT |  |  |  | 49 (25·4) | 12 (23·1) | 49 (26·3) |  | 93 (25·6) | 13 (32·5) | 4 (16·7) | 0 (0·0) |  |
| **α-thalassemia** |  |  |  | - | - | - | - |  |  |  |  |  |
| No α-thalassemia | 144 (44.9) | 49 (44.6) | 0.89 |  |  |  |  | 165 (45.5) | 13 (32.5) | 12 (50.0) | 3 (75.0) | 0.40 |
| Homozygous | 40 (12.1) | 12 (10.9) |  |  |  |  |  | 44 (12.1) | 4 (10.0) | 4 (16.7) | 0 (0.0) |  |
| Heterozygous | 137 (42.7) | 49 (44.5) |  |  |  |  |  | 154 (42.4) | 23 (57.5) | 8 (33.3) | 1 (25.0) |  |
| **G6PD deficiency** |  |  |  |  |  |  |  | - | - | - | - | - |
| No G6PD deficiency | 270 (84.1) | 93 (84.6) | 0.34 | 165 (85.5) | 44 (84.6) | 154 (82.8) | 0.40 |  |  |  |  |  |
| Hemizygous | 27 (8.4) | 13 (11.8) |  | 12 (6.2) | 4 (7.7) | 8 (4.3) |  |  |  |  |  |  |
| Homozygous | 20 (6.2) | 4 (3.6) |  | 3 (1.6) | 0 (0.0) | 1 (0.5) |  |  |  |  |  |  |
| Heterozygous | 4 (1.3) | 0 (0.0) |  | 13 (6.7) | 4 (7.7) | 23 (1.4) |  |  |  |  |  |  |
| **HbA1c level,** (%) | 5.8 (5.3:6.2) | 5.6 (5.1:6.1) | < 0.001^5^ | 5.5 (5.1:6.0) | 5.9 (5.2:6.6) | 5.6 (5.1:6.1) | 0.04^5^ | 5.6 (5.1:6.1) | 5.3 (4.7:6.0) | 5.4 (5.0:6.0) | 5.2 (5.0:5.6) | 0.17 |
| No PD/DM | 143 (44.6) | 88 (80.0) | < 0.001^5^ | 110 (56.9) | 23 (44.2) | 98 (52.7) | 0.25 | 187 (51.5) | 25 (62.5) | 16 (66.7) | 3 (75.0) | 0.23 |
| PD/DM | 178 (55.5) | 22 (20.0) |  | 83 (43.1) | 29 (55.8) | 88 (47.3) |  | 176 (48.5) | 15 (37.5) | 8 (33.3) | 1 (25.0) |  |
| **2hrs OGTT,** (mmol/L) | 7.6 (7.0:8.7) | 7.9 (7.1:8.6) | 0.39 | 7.6 (6.9:8.6) | 8.1 (7.1:9.3) | 7.8 (7:8.9) | 0.13 | 7.7 (7.0:8.7) | 7.5 (6.8:8.0) | 8.6 (7.4:9.6) | 8.1 (7.2:9.0) | 0.03^6^ |
| No PD/DM | 170 (53.0) | 50 (45.5) | 0.17 | 104 (53.9) | 20 (38.5) | 96 (51.6) | 0.14 | 182 (50.1) | 28 (70.0) | 8 (33.3) | 2 (50.0) | 0.03^6^ |
| PD/DM | 151 (47.0) | 60 (54.5) |  | 89 (46.1) | 32 (61.5) | 90 (48.4) |  | 181 (49.9) | 12 (30.0) | 16 (66.7) | 2 (50.0) |  |
| SCT, sickle cell trait; G6PD, glucose 6-phosphate-dehydrogenase; PD/DM, prediabetes/diabetes; homozygous α^+^ AT, homozygous α-thalassemia; heterozygous α^+^ AT, heterozygous α thalassemia; G6PD(B), no G6PD deficiency; G6PD(A), heterozygous G6PD deficiency; G6PD(A-), hemizygous/homozygous G6PD deficiency. Hemizygous and Homozygous G6PD deficiency is for males and female respectively.  **^1^**Data are number (%) unless specifically indicated as mean (SD) or median (IQR).  ^2^Only female were heterozygous G6PD deficiency (G6PD(A)).  ^3^Participants with SCT and hemizygous G6PD deficiency had a lower body mass index compared to the normal participants.  ^4^Average hemoglobin of those with homozygous α –thalassemia was lower compared to those with no α-thalassemia (11.8 vs. 12.6 g/dl)  ^5^Participants with SCT had a lower mean HbA1c level compared to participants with no SCT (5.6 vs. 5.8 mmol/L) while those with homozygous α-thalassemia had a higher mean HbA1c compared to those with no α-thalassemia (5.9 vs. 5.6 mmol/L).  ^6^Mean 2-hrs OGTT level was higher for those with hemizygous G6PD deficiency compared to those with no G6PD deficiency (8.6 vs. 7.7 mmol/L) | | | | | | | | | | | | |
